# Acute Sepsis-Induced Cholestatic Disease Presenting With Isolated Hyper-Bilirubinemia

**DOI:** 10.7759/cureus.35378

**Published:** 2023-02-23

**Authors:** Nariman Hossein-javaheri, Omar Rafa, Alyssa Reese, Amera Alsalahi

**Affiliations:** 1 Internal Medicine, University at Buffalo, Buffalo, USA; 2 Otolaryngology, Jacobs School of Medicine and Biomedical Sciences, University at Buffalo, Buffalo, USA; 3 Internal Medicine, Jacobs School of Medicine and Biomedical Sciences, University at Buffalo, Buffalo, USA

**Keywords:** gastroenterology, hepatitis-a, severe sepsis, cholestatic liver disease, cirrhosis

## Abstract

Sepsis-induced cholestatic disease occurs in a fair amount of critically-ill patients. Although the mechanism is poorly understood, hypoperfusion to the liver is one of the most common mechanisms that lead to liver dysfunction and subsequently biliary disease. Hepatic conditions such as cirrhosis and hepatitis A may have an impact on how sepsis-induced cholestatic disease can present. Understanding the presentation of sepsis-induced cholestasis and addressing the underlying cause of sepsis can certainly lead to better outcomes without the need for procedure intervention. We explore a patient with acute sepsis-induced cholestatic disease who had recently-resolving hepatitis A infection and underlying cirrhosis.

## Introduction

Sepsis can lead to multi-organ dysfunction and eventually organ failure resulting in death [1]. The pathogenesis involves systemic vasodilation of blood vessels. This leads to the lack of perfusion of blood flow to organs. Without a blood supply, cells lack oxygen to perform basic metabolic functions required to sustain life. In 2019, over 200,000 sepsis-related deaths occurred in the United States, with the majority occurring in adults over the age of 65 [2]. 

Alterations in the production, transport, and excretion of bile secondary to sepsis can precipitate cholestatic liver disease [3]. An observational prospective study of 608 patients with sepsis found that only 10.9% were diagnosed with sepsis-associated cholestasis, while 89.1% had sepsis without cholestasis [4]. Furthermore, one study found cholestasis was present in 33% of patients with septic shock who had been admitted to an intensive care unit [5]. Sepsis-related cholestasis often presents with jaundice alongside conjugated hyperbilirubinemia with normal or mildly elevated liver enzymes and alkaline phosphatase (ALP) [3]. Improvements in jaundice and abnormal lab values often occur alongside sepsis resolution, supporting the notion that these two conditions transpire concurrently. 

Previous studies have demonstrated that having pre-existing liver diseases may correlate with sepsis-related hepatitis [6]. Thus, presentations can vary as a result of coexisting conditions [7].

## Case presentation

A 71-year-old male patient with a past medical history of liver cirrhosis due to hepatitis C requiring transjugular intrahepatic portosystemic shunt (TIPS) procedure, grade 1 diastolic heart failure, chronic obstructive pulmonary disease (COPD), and chronic venous stasis of both lower extremities, presented to the emergency department with weakness due to three days of unspecific gastrointestinal (GI) symptoms including nausea, vomiting, and watery diarrhea. On arrival, he was hemodynamically unstable. His body mass index was 34 kg x m^2. He denied recreational drug use, alcohol abuse, and tobacco use. On examination, he had scleral icterus, was jaundiced, and had crackles over the bilateral lung bases on auscultation. Jugular venous distention (JVD) was present at the level of the mandible. He had a non-tender but distended and tympanic abdomen. He also had bilateral lower extremity edema with non-healed wounds. Of significance, a leukocytosis of 13.2 x 109/L (normal range: 4.5 to 11.0 × 10^9/L), thrombocytopenia at 58,000 platelets (normal range: 150,000 to 450,000), total bilirubin at 4.9 mg/dL (normal range: 0.1 to 1.2 mg/dL), lactic acid of 6 mmol/L (normal range: 0.5 to 2.2 mmol/L)., serum creatinine of 2.5 mg/dL (normal range: 0.74 to 1.35 mg/dL), troponin of 0.05 ng/ml (normal range: 0 to 0.04), and an elevated brain natriuretic peptide (BNP) of 530 pg/ml (normal range: less than 100 pg/mL) were noticed. Obtained chest X-ray showed prominent left basilar airspace opacities and the bedside lung ultrasound showed B-lines. He was diagnosed with acute hypoxic respiratory failure due to septic shock. He received gentle hydration and was treated with Intravenous (IV) vancomycin and piperacillin/tazobactam. The patient underwent extensive infectious evaluation and was found to have *Escherichia coli *bacteremia likely from his lower extremity wounds, which were also positive for* E. coli *via peripheral skin swab. Antibiotic therapy was switched to ceftriaxone given microbial sensitivity. 

**Figure 1:**
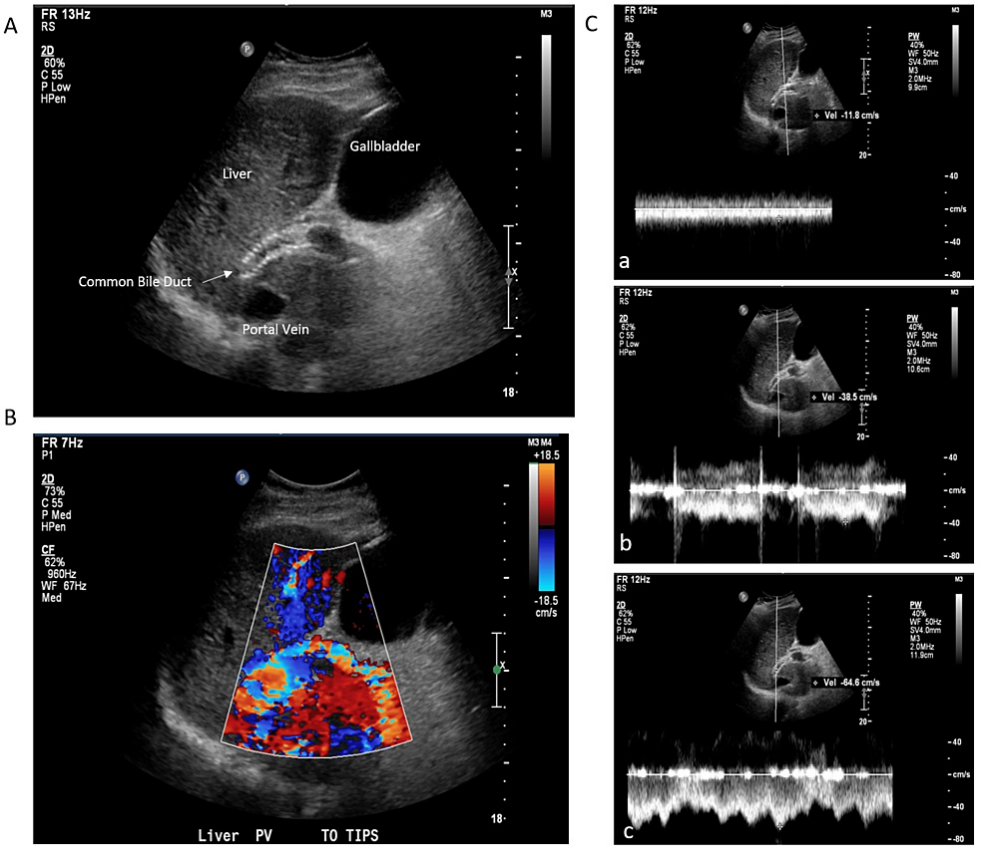
A: Right upper quadrant ultrasound,  B: Doppler ultrasound, C: TIPS flow rates (proximal (a), middle (b), and distal (c)) with shunt velocities in the proximal, middle, and distal stents measuring at 12, 39, and 65 cm/s, respectively. TIPS: Transjugular intrahepatic portosystemic shunt

**Figure 2:**
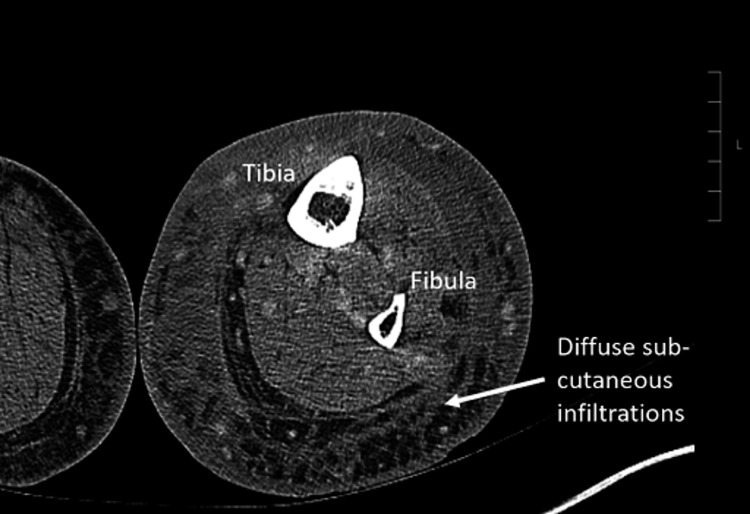
CT scan showing diffuse subcutaneous infiltration

Sepsis was treated with ceftriaxone daily for six days. Despite targeted therapy, leukocytosis and conjugated hyperbilirubinemia worsened. A right upper quadrant abdominal ultrasound was obtained showing liver cirrhosis and a common bile duct of 4mm. The TIPS was also evaluated and showed a normal direction of flow and waveforms within the portal and hepatic veins (Figure 1). Considering ceftriaxone might cause pseudolithiasis, antibiotic therapy was switched to cefazolin. For further investigation, a lower extremity CT scan was obtained showing diffuse subcutaneous infiltration and skin thickening without evidence of osteomyelitis, or drainable fluid collection from cellulitis (Figure 2). Poor response to initial therapy was attributed to inadequate antibiotic coverage and likely to undiagnosed methicillin-resistant *Staphylococcus aureus *(MRSA) cellulitis. The patient was started on vancomycin after which leukocytosis and hyperbilirubinemia both improved. A viral hepatitis panel was obtained and he was positive for hepatitis A IgG and IgM but negative for hepatitis B. Review of prior records showed positive hepatitis C IgG despite current serologies showing a negative result. Although a magnetic resonance cholangiopancreatography (MRCP) was considered, the patient was not a suitable candidate due to the history of the TIPS procedure. Given improvement and considering the patient’s desires, he was discharged on doxycycline and was scheduled for further follow-up with hepatology as an outpatient. 

Overall, it was concluded that the patient’s initial unspecific GI symptoms were due to hepatitis A infection and sepsis from *E. coli *bacteremia with *E. coli* and MRSA cellulitis. Cholestatic liver injury was attributed to cholestasis of sepsis. 

## Discussion

Although the overall incidence of cholestasis in critically ill patients seems to be around 33%, several etiologies couldd lead to it [5]. The two main mechanisms arise from bile acids and bilirubin uptake and transport impairment due to hypoxia and hypoperfusion, and/or due to inflammatory cytokines and endotoxins that negatively impact the function of the biliary cells and hepatocytes [8]. A combination of laboratory abnormalities in sepsis-induced cholestasis has been reported. Although alanine transaminase (ALT), aspartate transaminase (AST), and alkaline phosphatase (ALP) may be normal or elevated, conjugated bilirubin is always elevated which reflects intrahepatic cholestasis [8]. In this case report, we witnessed elevated hyperbilirubinemia and ALP with cholestasis of sepsis that improved with targeted antibiotic therapy. What makes this case more unique is the patient’s history of cirrhosis and concomitant hepatitis A infection.

In addition to sepsis, the following two factors may have played a role in this patient’s atypical presentation: the patient’s cirrhosis secondary to prior chronic hepatitis C infection, which required TIPS, and prior recent hepatitis A virus (HAV) exposure. The former demonstrates the patient’s liver’s lack of synthetic function which could have contributed to the presentation: normal liver function tests (LFTs) with isolated hyperbilirubinemia and elevated ALP. For example, one paper analyzed the poor diagnostic predictor of LFTs in patients with cirrhosis as a tool for acute liver injury showing that patients can have normal ALT [9]. With regards to the latter factor, the patient had HAV IgM antibodies (Ab) which signifies an infection of the virus within the past six months [10]. This validates the possibility of prior hepatic insult contributing to an atypical presentation of sepsis-related cholestasis. The notion that there could have been an ischemic component cannot be excluded because LFTs can be normal in patients with cirrhosis [11]. The reason here is that ischemia generally leads to LFTs trending up due to damaged hepatocytes releasing enzymes. However, a liver that has undergone cirrhosis changes will likely not produce any enzymes because of a lack of synthetic function due to chronic scarring. Hence, ischemic liver damage would not yield elevated LFTs. Regardless of predisposing factors, the treatment of underlying sepsis is the best approach in such patients and was proven to resolve our patient’s cholestasis.

Complications arising from sepsis-induced cholestatic liver injury include eventually fulminant liver failure if sepsis is not treated. The goals of treatment, as discussed above, include treating the underlying cause of sepsis. In our case, we treated the patient's bacteremic infection. The prognosis is generally dependent on a case-to-case basis. Of importance, if the patient is continuously requiring IV fluid support, vasopressors, and artificial airway support, this is a sign of poor prognosis. 

## Conclusions

Sepsis-induced cholestasis disease can present as normal LFTs with isolated hyper-bilirubinemia in patients with cirrhosis or acute infective hepatitis. Many cases can be multifactorial. It is important for clinicians to understand the collective role of sepsis, hepatic ischemia, and concomitant viral infections such as HAV in bile transport dysfunction. Management can be pursued conservatively by addressing the underlying cause of sepsis without the need for invasive interventions.
